# The Genomic Context for the Evolution and Transmission of Community-Associated *Staphylococcus aureus* ST59 Through the Food Chain

**DOI:** 10.3389/fmicb.2020.00422

**Published:** 2020-03-17

**Authors:** Rui Pang, Shi Wu, Feng Zhang, Jiahui Huang, Haoming Wu, Junhui Zhang, Yanping Li, Yu Ding, Jumei Zhang, Moutong Chen, Xianhu Wei, Youxiong Zhang, Qihui Gu, Zhenwen Zhou, Bingshao Liang, Wenzhi Li, Qingping Wu

**Affiliations:** ^1^State Key Laboratory of Applied Microbiology Southern China, Guangdong Provincial Key Laboratory of Microbial Culture Collection and Application, Guangdong Open Laboratory of Applied Microbiology, Guangdong Institute of Microbiology, Guangdong Academy of Sciences, Guangzhou, China; ^2^Department of Food Science and Technology, Jinan University, Guangzhou, China; ^3^Clinical Laboratory, Guangzhou Women and Children’s Medical Center, Guangzhou Medical University, Guangzhou, China; ^4^Infinitus (China) Company Ltd., Jiangmen, China

**Keywords:** *Staphylococcus aureus*, molecular evolution, ST59 lineage, transmission, food chain

## Abstract

Sequence type 59 (ST59) is a predominant clonal lineage of community-acquired, methicillin-resistant *Staphylococcus aureus* (CA-MRSA) in Asia. Despite its increasing clinical relevance in China, the evolution and geographic expansion of ST59 has been relatively uncared for. Previous study has shown that ST59 was the predominant clone in food-related MRSA in China. This study compared the genomes of 87 clonal complex (CC) 59 *S. aureus* isolates sourced from food chain and infection cases to reconstruct the molecular evolution and geographical spread of ST59. Accordingly, three major sub-clades of ST59 were identified and these did not correlate with isolation source or location. Phylogenetic analysis estimated that ST59 in mainland China diverged from a most common recent ancestor around 1974, and most of the cases of cross-country transmission occurred between 1987 and 2000. Notably, two recent events of cross-country transmission through the food chain were observed, the isolates from these events diverged within relatively short time intervals. These isolates also showed high similarity in terms of their core genome, accessory genes, and antibiotic resistance patterns. These findings provide a valuable insight into the potential route of ST59 expansion in China and indicate a need for robust food chain surveillance to prevent the spread of this pathogen.

## Introduction

*Staphylococcus aureus* is an opportunistic pathogen that causes serious community- and hospital-acquired disease; it accounts for the majority of skin and soft tissue infections in humans, and is also a causative agent of infective endocarditis, necrotizing pneumonia, septicemia, and toxic shock syndrome (Lowy, 1998; Diep et al., 2006). Particularly concerning is the increasing incidence of methicillin-resistant *Staphylococcus aureus* (MRSA) infections, which have emerged as a major public health concern. Indeed, MRSA was once considered to be solely healthcare-acquired (HA-MRSA), yet over the past few decades, it has increasingly been identified in community-acquired infections (CA-MRSA) (David and Daum, 2010).

Interestingly, CA-MRSA isolates primarily belong to a subset of clonal lineages and possess specific staphylococcal cassette chromosome *mec* (SCC*mec*) elements, which confer methicillin resistance, and the phage-encoded toxin, Panton-Valentine leukocidin (PVL), which contributes to skin and soft tissue infections (Otto, 2013). Currently, five major CA-MRSA clones have been described and are associated with specific geographic areas, sequence type (ST)1, ST8, ST30, ST59, and ST80 (Mediavilla et al., 2012; Chuang and Huang, 2013; Abdulgader et al., 2015). In the Asia-Pacific region including Australia, ST59 predominates, where it accounts for 56% of pediatric CA-MRSA infections in Taiwan (Huang et al., 2008; Chuang and Huang, 2013), and has been associated with an increasing proportion of MRSA infections in mainland China (Li et al., 2018; Liang et al., 2019). In Hong Kong, a recent outbreak of ST59 MRSA has occurred in a neonatal intensive care unit (Cheng et al., 2019). Li et al. (2018) indicated that ST59 possesses a fitness advantage over other MRSA clones and that it poses a critical threat to human health. However, the evolution and expansion of *S. aureus* ST59 in this region has been relatively understudied.

Previous studies have shown that *S. aureus* ST59 harbors two major clones: the Taiwan clone (TW), which causes severe infections, and the Asian-Pacific clone (AP), which is typically commensal (Huang et al., 2008). Genomic comparisons have revealed that the TW clone carries a PVL-encoding prophage ΦSa2 (Chen et al., 2013), whereas the AP clone carries a staphylokinase (SAK)-encoding prophage ΦSa3 that enhances the bacterium’s capacity to colonize human hosts (van Wamel et al., 2006; Kwiecinski et al., 2013). Thus, the origins of most ST59 isolates can be determined based on the presence of either PVL- or SAK-related phage. However, a minority of publicly available ST59 genomes indicate the presence of both phages (Feng et al., 2017), which suggests that MRSA ST59 may have multiple origins or that it has experienced recombination events during its evolution and genetic expansion.

Though little is known about the transmission route of the ST59 lineage, a previous study completed by our research group documented the prevalence of this *S. aureus* clone in various food samples across China (Wu et al., 2019). Indeed, 52.8% of MRSA isolates collected from various food sources were identified as clonal complex (CC)59, which includes ST59, ST338, and ST3355, and suggests that the food chain may serve as a potential transmission vector. However, zoonotic MRSA infections are primarily livestock-associated (LA-MRSA) (Petinaki and Spiliopoulou, 2012), and these are not typically members of CC59. For example, LA-MRSA isolates from Europe and North America primarily belong to CC398, and the vast majority of LA-MRSA isolates from Asia belong to CC9 (Bens et al., 2006; Cui et al., 2009; Smith et al., 2009; Ye et al., 2016). Indeed, CC59 isolates are rarely linked to LA infections but are frequently associated with CA infections. Thus, we hypothesized that the prevalence of *S. aureus* ST59 in foods is due to human activity that spreads the bacterium through the food chain.

To characterize the potential transmission of *S. aureus* ST59 in the food chain, we compared the whole-genome sequences of 81 CC59 isolates from geographic areas across China. Using a Bayesian temporal and spatial analysis, we estimated the evolutionary properties of this *S. aureus* clone during its geographic expansion in China. Two potential food chain transmission events were identified, revealing that the ST59 lineage may have spread across the different regions using food as a vehicle. These findings are critical to understand the evolutionary signatures that correlate with the expansion of this pathogen.

## Materials and Methods

### Selection of Bacterial Isolates

From July 2011 to June 2016, we collected 4,300 retail food samples from supermarkets, fairs and farmers’ markets covering most of the provincial capitals of China (Supplementary Figure S1). These samples included 604 raw meat (bacon/sausage, poultry, pork, mutton, and beef), 860 aquatic products (freshwater fish, shrimp, and seafood), 601 quick-frozen products (frozen dumplings/steamed stuffed buns and frozen meat), 859 ready-to-eat (RTE) food, 258 pasteurized milk, 419 vegetables, and 699 edible mushrooms. A total of 1,581 *S. aureus* isolates were obtained from 1,063 positive samples according to the GB 4789.10-2010 food microbiological examination of *S. aureus* (National Food Safety Standards of China) and the most probable number (MPN) method (Gombas et al., 2003). They were identified by Gram stain, catalase and oxidase tests and API STAPH test strips (BioMerieux, Marcy-1’Etoile, France). According to a multilocus sequence typing (MLST) analysis, 68 typical *S. aureus* CC59 isolates were further selected (Supplementary Table S1). The presence of the *mecA*/*mecC* gene was assessed by PCR for all isolates (Perez-Roth et al., 2001; Stegger et al., 2012). Nineteen additional ST59 isolates that had been previously sequenced were retrieved from the NCBI database for comparative genomic analysis (Supplementary Table S1).

### Whole-Genome Sequencing and Assembly

Genomic DNA was extracted from *S. aureus* isolates using a genomic DNA extraction kit (Magen Biotech, Guangzhou, China) according to the manufacturer’s instructions. Each DNA sample was fragmented into 400-bp fragments by a Covaris M200 sonicator and prepared for sequencing with the Ion Plus Fragment Library Kit (Thermo Fisher Scientific Inc., Waltham, MA, United States). Whole genomes were sequenced on the Life Ion S5 platform with an average coverage of 100×. Clean reads were used for *de novo* assembly with SPAdes v3.6.2 (Bankevich et al., 2012). Only assemblies that harbored ≥95% of core genome (cg) MLST targets were used for further analysis as previously described (Strauß et al., 2017). If the criteria were not met, the sample was re-sequenced. The presence of PVL- and SAK-coding genes was determined using BLAST.

### Single Nucleotide Polymorphisms Calling in Core Genomes and Phylogenetic Inference

The core genome of the ST59 clone was produced using Harvest v1.1.2 using the SA40 genome as a reference (Treangen et al., 2014). After the core-genome alignment was generated, Gubbins was used to conduct recombination analysis and remove the putative recombined regions (Croucher et al., 2015). Single Nucleotide Polymorphisms (SNPs) were then extracted from the recombination-free core-genome alignment using the script available at https://github.com/sanger-pathogens/snp-sites. The ML phylogenetic tree was constructed on the concatenated core SNPs using RAxML v8.2.10 in the GTRGAMMA model (1,000 bootstrap) (Stamatakis, 2014), and was visualized using iTOL (Letunic and Bork, 2016).

### Time-Scaled Phylogenetic Analysis

To explore the transmission relationship within ST59 isolates, we inferred the substitution rate, divergence dates, and phylogeographic spread, according to the Bayesian methods implemented in BEAST v1.8.4 package (Drummond et al., 2012). The best nucleotide substitution model was calculated by jModelTest v2.1.7 (Darriba et al., 2012). The core SNP alignment was then used for Bayesian inference with the general time-reversible (GTR) model (inferred best model) of nucleotide substitution, and the symmetric substitution model of discrete trait substitution with the option to perform Bayesian Stochastic Search Variable Selection (BSSVS) selected. Different molecular clock (strict, random, and uncorrelated lognormal) and tree (constant size, exponential growth, Bayesian skyride, and Bayesian skyline) models were run. Each model was run twice for 100,000,000 states, with a sampling frequency of 10,000, to check for convergence in the data set. The convergence of all model combinations was checked using Tracer module in BEAST (threshold: ESS of the tree model over 200), and the maximum clade credibility (MCC) tree was generated with the TreeAnnotator according to the best model (strict molecular clock and constant size model in this study) and visualized with FigTree 1.4.3.

### Pan-Genome Analysis

The genomes of all analyzed isolates were annotated using Prokka v1.11 (Seemann, 2014). The output of Prokka was used to construct the pan-genome using Roary v3.11.2, with a BLASTP identity cutoff of 90% (Page et al., 2015). A profile of absence (0) or presence (1) of all genes was generated according to the pan-genome distribution of all samples, and a hierarchically clustered heatmap was created with this matrix by using the Pearson correlation function in R (Graña-Miraglia et al., 2017).

### Average Nucleotide Identity Calculation

Pairwise ANIs between all isolates were calculated on whole genome sequences by using the Python module Pyani v0.2.8 with default parameters^1^. The Average Nucleotide Identity (ANI) result was presented as a heatmap generated according to a matrix of Hadamard product of pairwise alignment coverage, total alignment lengths, similarity errors, and percentage identity; hierarchical clustering in two dimensions was represented by dendrograms constructed by simple linkage of the data (Pritchard et al., 2016).

### Assessment of Antibiotic Resistance Patterns

Antimicrobial susceptibility tests were performed using the agar disk diffusion method (Kirby–Bauer) on Mueller–Hinton agar for all of the *S. aureus* isolates. The antimicrobial agents used for susceptibility testing were amoxicillin/clavulanic acid, ampicillin, cefepime, cefoxitin, penicillin G, ceftazidime, amikacin, gentamicin, kanamycin, streptomycin, chloramphenicol, clindamycin, erythromycin, telithromycin, ciprofloxacin, norfloxacin, tetracycline, linezolid, trimethoprim/sulphamethoxazole (1:19), rifampicin, quinupristin/dalfopristin, teicoplanin, nitrofurantoin, and fusidic acid. MICs for linezolid-resistant isolates by disk diffusion were also confirmed by the agar dilution method on Mueller–Hinton agar. The results were scored and graded as resistant (R) or sensitive (S) according to the guidelines of The Clinical and Laboratory Standards Institute [CLSI] (2015). *S. aureus* ATCC25923 and *Escherichia coli* ATCC25922 were included as controls. The pairwise correlation value between each two isolates was estimated using the software available in OmicShare^2^ based on the Pearson correlation coefficient.

### Spatial Phylogenetic Reconstruction

SpreaD3 v0.9.7.1 was used to analyze and visualize phylogeographic relationships resulting from the Bayesian inference of sequences and spatiotemporal diffusion (Bielejec et al., 2016). Here we mapped phylogenies annotated with discrete spatial information on the genomes of ST59 clones generated by the BSSVS procedure. High-dimensional posterior summaries to markup the spatial diffusion through time were identified using a Bayes Factor test (cut-off ≥10).

### Data Accessibility

The draft genome sequences of *S. aureus* generated in this study were submitted to the NCBI database under BioProject PRJNA589244.

## Results

### Phylogenomic Analysis of the ST59 Lineage

We performed whole genome sequencing on 63 isolates of ST59, four ST338, and one ST3355 isolated from food samples across China (Supplementary Table S1). Of these, 45 isolates were methicillin-resistant, and 23 were methicillin-susceptible *S. aureus* (MSSA). According to *spa* typing, most of these isolates were *spa* type t437 (84.3%), as was previously described by Li et al. (2018). Additionally, 19 publicly available *S. aureus* ST59 genomes were included in the comparative analysis (Supplementary Table S1). Importantly, these strains were also isolated in Asia.

**FIGURE 1:**
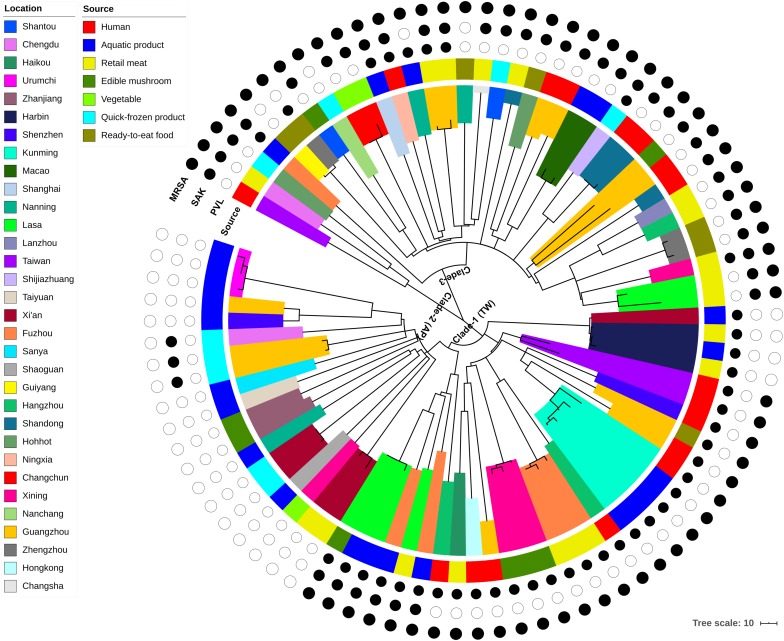
Maximum-likelihood phylogeny of 87 CC59 *S. aureus* isolates based on 3,745 core genome single nucleotide polymorphisms. The clade colors indicate each isolates region of origin, and the bar color indicates the source of each isolate. Information about the presence (black circle) or absence (white circle) of PVL and SAK is given for each isolate. Scale bar: SNP distance.

Phylogenomic analyses were carried out using the combined dataset of 87 genomes, which included isolates from humans (*n* = 17), aquatic products (*n* = 24), retail meats (*n* = 19), quick-frozen products (*n* = 9), RTE food (*n* = 7), edible mushrooms (*n* = 8), and vegetables (*n* = 3). Only two putative recombination regions were identified, which both occurred on the branch of isolate AR3 (Supplementary Table S2). After the removal of recombination events, these isolates differed in 3,745 core genome SNPs. The concatenated core SNPs were used to construct a maximum-likelihood (ML) phylogenetic tree (Figure 1).

According to the node height, the ST59 lineage was divided into three major clades. The clade 1 and clade 2 shared common ancestors with the TW and AP clones respectively, while the clade 3 was genetically distinct from these two widely reported clones. Notably, there was no significant correlation between clades and source or location when the geographic and source origin of isolates were mapped onto the phylogenetic tree. While this revealed the frequent transmission of ST59 clones between geographic areas and food sources, it also suggested that no significant boundary existed between human-infected and food-contaminated isolates. For example, the isolates collected from aquatic products in Kunming were very closely related to another clinical isolate collected in this region (clade marked by cyan in Figure 1). The presence or absence of PVL- and SAK-coding genes was also assessed, as these markers have previously been used to distinguish the origins of ST59 clones (Chen et al., 2013). As such, approximately 78% of isolates carried PVL or SAK, 15% carried both PVL and SAK, and the remainder were negative for PVL and SAK (Figure 1 and Supplementary Table S1). The presence of PVL and SAK in isolates in clade 1 and clade 2 was largely consistent with the previous reports concerning TW (PVL positive and SAK negative) and AP (PVL negative and SAK positive) clones. However, clade 1 has become more diverse as it spread across China, with two potential introductions of SAK. The existence of separate PVL-negative and -positive MSSAs in this clade also indicates that one introduction of PVL and a subsequent loss, and a single introduction of SCC*mec* may have occurred. For clade 3, repetitive introductions and losses of PVL were observed, indicating that this clade may present more variability in the genomic regions of these genes.

### Cross-Country Transmission of the ST59 Lineage

To further reconstruct the evolution and geographic transmission of the ST59 lineage, the core SNPs were used for Bayesian phylogenetic analysis with BEAST. The substitution rate in the core genome of the ST59 lineage was estimated to be 1.31 × 10^–6^ substitutions per site per year [95% highest posterior density (HPD) interval = 9.89 × 10^–7^ to 1.62 × 10^–6^], corresponding to 2.80 SNPs per year (95% HPD = 2.12–3.46). This substitution rate agreed with previously published data for other *S. aureus* lineages and revealed a relative conservation of the mutation rate across *S. aureus* lineages (McAdam et al., 2012; Uhlemann et al., 2014, 2017; Moradigaravand et al., 2017).

**FIGURE 2:**
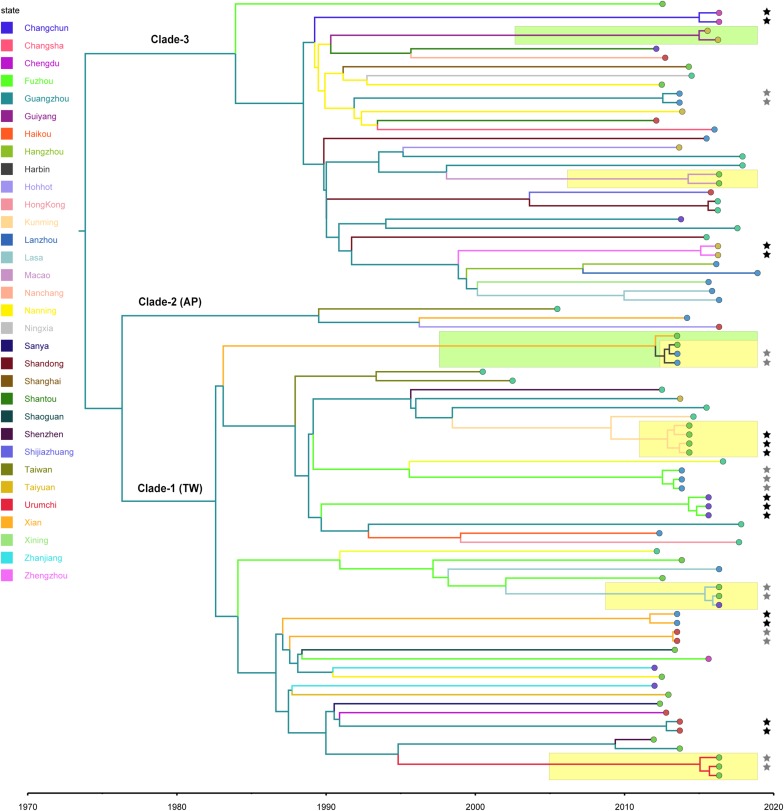
Bayesian maximum clade credibility tree of CC59 isolates. Branch colors represent the region of origin of each sample and their most recent common ancestor, respectively. The color of the circle at the end of each branch indicates the isolate’s source; cyan represents isolation from a human, green from an aquatic source, blue from retail meat, gold from an RTE food, red from a quick-frozen product, purple from an edible mushroom, and orange from a vegetable. Isolates that were source from the same food sample are marked up with colored stars behind the tree tips. Clades that were estimated to have been involved in recent local transmission are highlighted with yellow squares, and those estimated to have been involved in recent cross-country transmission are highlighted with green squares.

Using the calculated substitution rate, the date of divergence of the ST59 lineage was estimated according to a MCC tree generated using TreeAnnotator. As such, the estimated time of the most recent common ancestor (MRCA) was approximately 1974 (95% HPD, 1963–1983); the MRCA originated in Guangzhou and diverged into three clades (Figure 2). These clades were then transmitted from Guangzhou to other cities and diverged in different directions. Notably, the ancestors of representative isolates for TW (SA957) and AP (SA40) clones were estimated to be transmitted from Guangzhou to Taiwan around 1988 (95% HPD, 1981–1994) and 1990 (95% HPD, 1983–1995), respectively. Further, an estimated 82% of spread events occurred between 1987 and 2000, which is also the period during which most of the cross-country ST59 transmissions occurred. This indicates that most of the recent food-borne cases of *S. aureus* ST59 were locally transmitted.

Notably, two relatively recent cases of cross-country transmission were observed (marked by green in Figure 2, also see Supplementary Figure S2 for details). Isolates 2939 and fhich were collected from Guiyang in July 2015 and from Zhengzhou in April 2016, respectively, diverged from a common ancestor originating in Zhengzhou in January 2015 (95% HPD, April 2014 to August 2015). These isolates were both identified in RTE foods; thus, RTE foods may have been responsible for the transport of the bacterium from Zhengzhou to Guiyang. Similarly, two aquatic isolates, 1931-0 and 1956-2, which were isolated in Xi’an and Harbin, respectively, in July 2013, shared a recent common ancestor in Xi’an in January 2012 (95% HPD, January 2011 to December 2012). Notably, this sub-clade was also isolated from aquatic products and retail meat in Harbin, which is likely the result of cross-contamination in this region.

**FIGURE 3:**
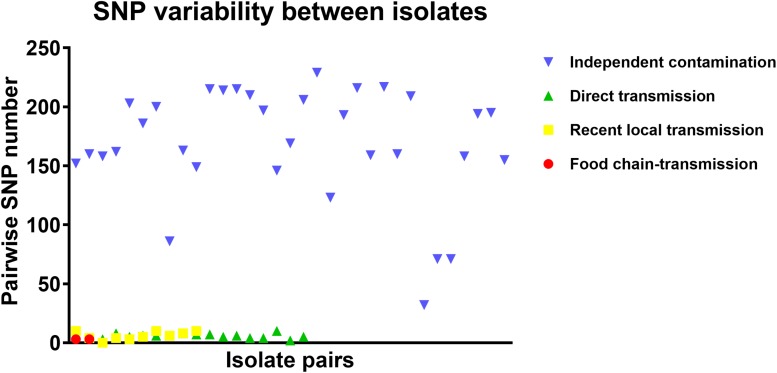
The pairwise SNP comparison between *S. aureus* isolates from different case types.

To further characterize the putative food chain-transmitted cases, the pairwise SNP distances between isolates were estimated. Excluding those distinct isolates, paired isolates collected from the same sample (direct transmission, marked up with colored stars behind the tree tips) differed by six SNPs (range: 2–10; Supplementary Table S2 and Figure 3). Isolate pairs collected from the same region with shorter divergence time (<2 years, recent local transmission, marked by yellow bar in Figure 2) were also closely related, with an average pairwise SNP distance of six (range: 0–10). In contrast, longitudinal isolates collected from the same region and separated by time interval more than 2 years (independent contamination) harbored a considerably higher average pairwise SNP distance of 169 (range: 32–229). The pairwise SNP distances between isolates 2939 and 1956-2 and their counterparts were both three, indicating that these isolates could be precisely linked to recent transmissions.

### Genetic and Phenotypic Evidence for Food Chain Transmission Events

**FIGURE 4:**
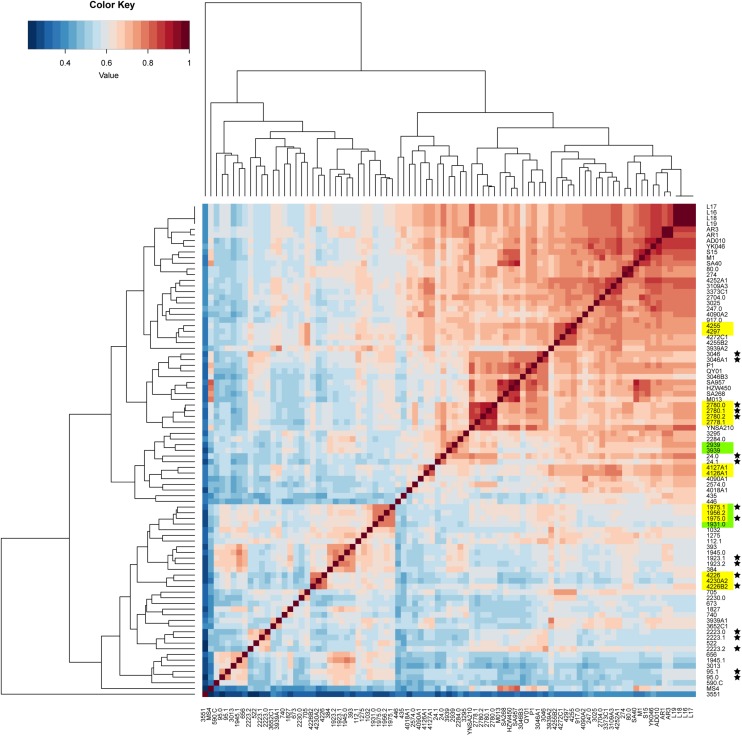
Comparison on the accessory gene profiles among the isolates. The differences in accessory gene contents are presented by a heatmap of the gene content correlation matrix. The dendrograms across the top and side indicate the hierarchical clustering by gene content. Isolates that were source from the same food sample are marked up with colored stars. Isolates estimated to have been involved in recent local transmission are marked with yellow, and those estimated to have been involved in recent cross-country transmission are marked with green.

The pan-genome was analyzed to determine whether the putative food chain-transmitted isolates shared similar accessory genome by virtue of their relatively short timescale over which they diversify from a common ancestor. A gene content correlation matrix was constructed according to the profile of accessory gene presence and absence, and was visualized using a heatmap (Figure 4). The dendrogram shown in the heatmap was not fully correlated with the phylogenetic tree inferred from core genome SNPs. However, most of the isolates from direct transmission and recently local transmission still clustered together as was shown in the phylogenetic tree. Further, the clustering of the putative food chain-transmitted isolates is rather similar to the topology of phylogenetic tree. Hence, the similarity between phylogenetic relationships and clustering profiles gathered from gene content variation might due to the short divergence time between these isolates.

The pairwise ANI was calculated based on the whole genome sequences of all the isolates, and a dendrogram tree was constructed based on the clustering of the pairwise ANI values (Supplementary Table S3 and Supplementary Figure S3). This analysis takes into account the core and accessory genomes of each isolate. As such, the clustering of the putative food chain-transmitted isolates was highly correlated to the topological patterns inferred from core genome SNPs and accessory gene contents. Thus, the genetic similarities between these isolates and their counterparts are likely the result of relatively recent divergences.

**FIGURE 5:**
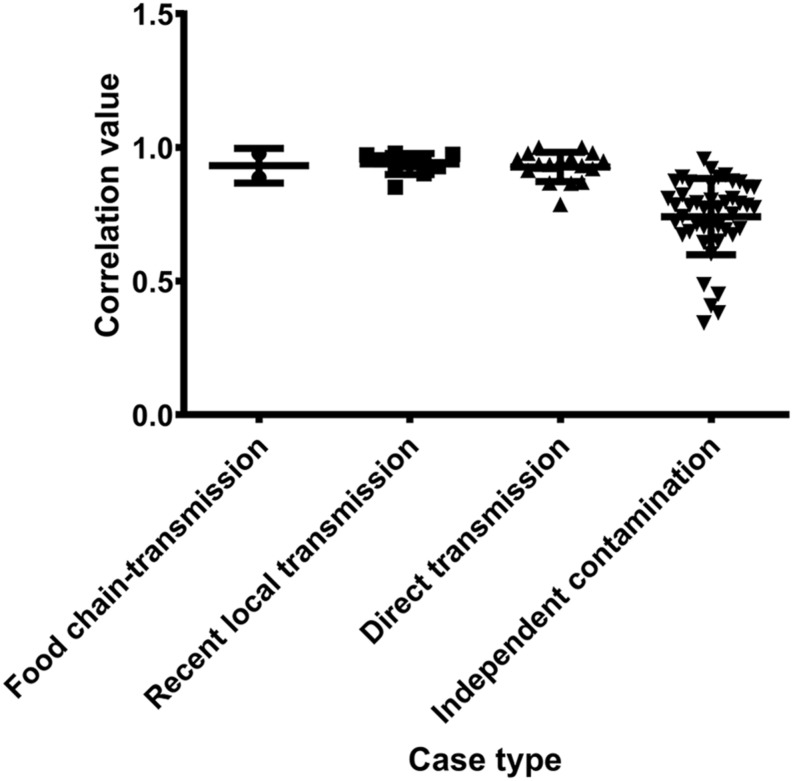
The pairwise correlation of antibiotic resistance patterns in *S. aureus* isolates between different case types.

Antibiotic resistance profiles were also considered of putative food chain-transmission, direct transmission, recently local transmission, and independent contaminated case isolates (Supplementary Table S4) and the pairwise correlation was calculated. According to the correlation value, the similarity in antibiotic resistance pattern of paired isolates within direct transmission or recent local transmission was much higher than that of paired isolates collected from independent contamination (Figure 5 and Supplementary Table S5). The resistance profiles were also highly similar between isolates 2939 and 3939 (correlation value 0.9773), and between isolates 1956-2 and 1931-0 (correlation value 0.8856), reinforcing the inference that the divergence time between these isolates and their counterparts was relatively short. Furthermore, these remarkably similarity in phenotypes unveiled a urgent consideration for public health that the antibiotic resistance would probably disseminate through bacterial transmission.

**FIGURE 6:**
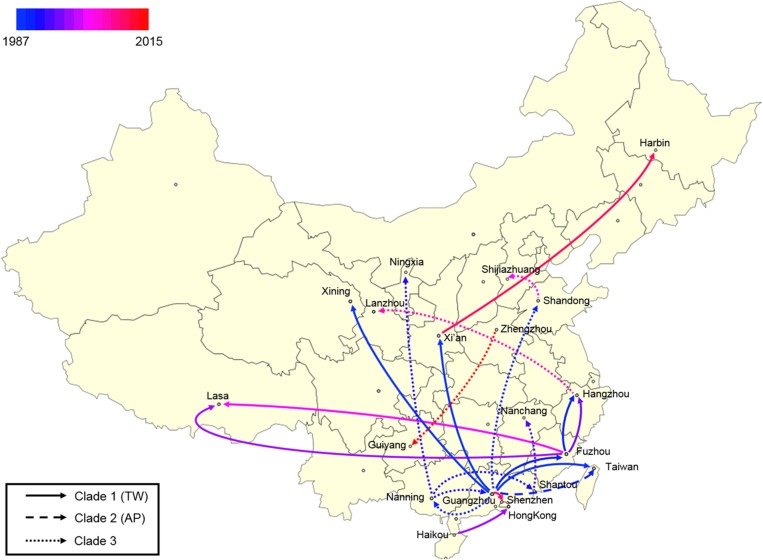
Spatial phylogenetic reconstruction of ST59 transmission dynamics inferred using SpreaD3. A Bayesian phylogeographic reconstruction incorporating discrete spatial spatiotemporal diffusion was used to visualize the transmission between the original regions of ST59 clones and the geographic locations where different lineages were detected throughout the expansion across China. Different linear types indicate that the transmission events fall within different phylogenetic clades.

Finally, a spatial phylogeographic reconstruction was used to visualize the potential transmission between geographic locations (Figure 6). As such, a Bayes factor cut-off of 10 produced a map that described several waves of ST59 transmission across China. As was estimated above, most transmission events occurred before 2000, and southern China was the most common transmission source of this clone to other regions. Based on the phylogeographic analysis, only two recent transmission events were identified: Zhengzhou to Guiyang in 2015, and Xi’an to Harbin in 2012. Considering that the food sources of contamination at both the departure and destination sites were identical in both events, the food chain may be a considerable reservoir for cross-country transmission of *S. aureus* ST59 in China.

## Discussion

The dissemination of MRSA is a worldwide problem; it is transmitted by humans in both the hospital and community settings, animals in the wild, or by vectors, such as air and water (Diekema et al., 2001; Goodwin et al., 2012; Porrero et al., 2013; Bos et al., 2016). However, most of these transmission pathways lack regularity, making it difficult to determine the origin of the MRSA clone, especially when clones have spread across the country. As a traceable vector, the food chain poses a significant risk for MRSA transmission across geographic regions, as it has been demonstrated by the illegal introduction and commerce of MRSA-contaminated food (Oniciuc et al., 2015; Rodriguez-Lazaro et al., 2017). Thus, the aim of this study was to characterize the evolutionary dynamic and transmission potential of MRSA through the food chain.

This study utilizes ST59 isolates that were isolated from the food chain across China (Wu et al., 2019). Previous studies have demonstrated that ST59 is becoming the predominant MRSA sequence type isolated from hospitalized patients in China (Chuang and Huang, 2013; Yang et al., 2017; Li et al., 2018). There are two distinct clones within the ST59 sequence type, AP, which is PVL-negative/SAK-positive, and TW, which is PVL-positive/SAK-negative (Hung et al., 2016). Both of these clones were identified in our analysis (Figure 1); however, both clones underwent significant genetic changes. In particular, the TW clone acquired the SAK-encoding phage twice, and lost the PVL-encoding phage during its spread between different regions in China. A clone (clade 3) was also identified that was genetically distinct from either the AP or TW clones. This clone showed higher instability in the PVL- and SAK-encoding phages than the other two clones. The variability of these phage genes was also shown in another MRSA linage USA500 (Frisch et al., 2018), unveiling the potential importance of horizontal gene transfer in evolution of pathogenic MRSA.

Several potential direct transmission cases were also identified in the dataset, including local and cross-country transmission. Generally, if two bacterial isolates are involved in a case of direct transmission, they share a low genome-wide SNP distance due to the limited divergence time (Uhlemann et al., 2014). In studies tracing the transmission of CA-MRSA in United States communities, the maximum pairwise distance of isolates within a single household in New York was determined to be 23 SNPs (Uhlemann et al., 2014), and the mean genetic diversities within Los Angeles and Chicago were 17.6 and 12 SNPs per household (Alam et al., 2015). In our study, the maximum pairwise SNP distance between paired isolates collected from the same sample was 10. As such, for isolates involved in putative local transmission, the pairwise SNP distances ranged from 0 to 10, which precisely fell within this range. Both of the putative cross-country transmission cases, isolate 2939 with 3939, and isolate 1956-2 with 1931-0, also harbored pairwise SNP distances lower than 10. The identical sources for these isolates, RTE foods for both 2939 and 3939, and aquatic products for both 1956-2 and 1931-0, reinforced the likelihood of direct transmission between these samples.

Given that the SNP-based analysis was focused on the core genes of these isolates, we also constructed a dendrogram according to the presence and absence of accessory genes. Importantly, the distribution of isolates between the different clades did not fully align with their presentation in the SNP-based phylogenetic tree; although other groups have reported similar discrepancies (Orsi et al., 2011; Anastasi et al., 2016; Graña-Miraglia et al., 2017; Henri et al., 2017). Several reasons may account for this observation, such as natural factors including horizontal gene transfer and recombination, and technical factors including gaps in the draft genomes and inaccurate parameters for the pan-genome estimation (Orsi et al., 2011; Rouli et al., 2015). Despite the discrepancy, most isolates that were estimated to be derived from direct transmissions grouped together, indicating that fewer variations in gene content had occurred between these isolates. The genetic relationships within these isolates was further validated by genome-wide ANI analysis, which considered both core and accessory genes (Pritchard et al., 2016).

Furthermore, this study demonstrated that *S. aureus* ST59 underwent a series of geographic expansions across China during the 1980s and 1990s (Figure 2) and that southeast coastal cities were the most frequent origins of the spread of this bacterial clone. A previous review has indicated that ST59 lineage mainly spreads along the Asia-Pacific including the southeast coastal of China (Chuang and Huang, 2013), which might exactly explain why *S. aureus* ST59 in mainland China were originated from this region. Importantly, this also aligns our previous findings, namely that ST59 was the predominant genotype among all MRSAs isolated from aquatic products in China (Wu et al., 2019). Thus, Guangzhou, a city known for consumption of large amounts of aquatic products, had been one of the major distributed cities for ST59 MRSA (Li et al., 2018). In addition, the dominant ST of *S. aureus* isolated from patients in Guangzhou was exactly belonged to CC59 (Liang et al., 2018). Our analysis also revealed Fuzhou as a hotspot for ST59 transmission. Actually, previous studies has showed the high incidence of ST59 infections in the pediatric population in Taiwan (Huang et al., 2008). The geographical adjacency of Fuzhou to Taiwan suggests that bacterial dissemination may have more frequently occurred through environmental vectors, such as sea water and migratory wildlife (Abulreesh, 2011; Beleneva, 2011; Robb et al., 2013). However, the focus of this study was ST59 food chain isolates, and further studies are necessary to accurately characterize the transmission of ST59 between these two sites.

In summary, the potential for cross-country transmission of *S. aureus* ST59 through the food chain is supported by this study, which facilitates an increased understanding of the evolution and geographic expansion of CA-MRSA. Given that the food chain is indispensable to human society, the assumption that contaminated food products may serve as vehicles for the spread of pathogens has been widely recognized (Ogata et al., 2012; Schoder et al., 2015; Oniciuc et al., 2017). However, most of the previous studies regarding MRSA in the food chain have only characterized the relationship between multiple bacterial isolates using multi-locus sequence typing (MLST) (Ge et al., 2017; Rodriguez-Lazaro et al., 2017; Rong et al., 2017; Tang et al., 2017; Wu et al., 2019), a method that is dependent on housekeeping genes. Thus, the genetic similarity of housekeeping genes cannot exclude the possibility that these isolates are derived from ancestors separated by earlier divergence events rather than by direct food chain-transmission. This study described a genomic approach to accurately determine the relationship between pathogens isolated from multiple geographic locations, rendering a more accurate system for monitoring and tracking foodborne pathogens.

## Data Availability Statement

The datasets generated for this study can be found in the NCBI database under BioProject PRJNA589244.

## Author Contributions

RP, SW, and QW conceived and designed the study, and wrote and finalized the manuscript. SW, FZ, JH, YD, JMZ, MC, XW, YZ, QG, ZZ, BL, and WL performed the samples and data collection. RP, JHZ, and YL conducted the genome sequencing. RP, SW, and HW performed the data analysis.

## Conflict of Interest

WL is employed by the company Infinitus (China) Company Ltd., Jiangmen, China. The remaining authors declare that the research was conducted in the absence of any commercial or financial relationships that could be construed as a potential conflict of interest.

## Footnotes

1 https://github.com/widdowquinn/pyani

2 http://www.omicshare.com/
