# Supplementary figures and images for: The Genomic Context for the Evolution and Transmission of Community-Associated Staphylococcus aureus ST59 Through the Food Chain
Source: Front Microbiol. 2020 Mar 17;11:422. doi: 10.3389/fmicb.2020.00422 (PMC7090029; doi:10.3389/fmicb.2020.00422)

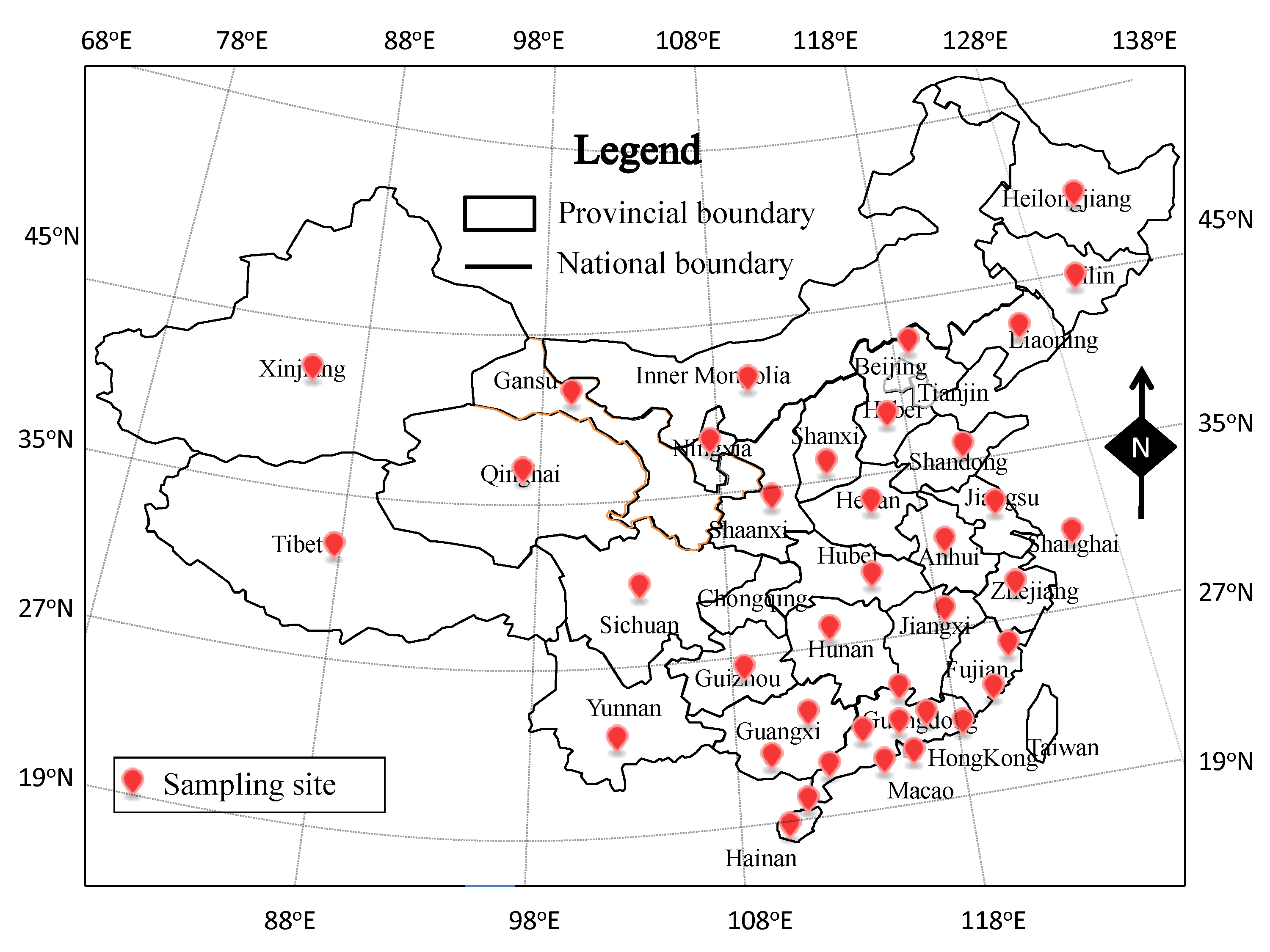

Supplement: Supplementary file 1 [file Image_1.TIF]

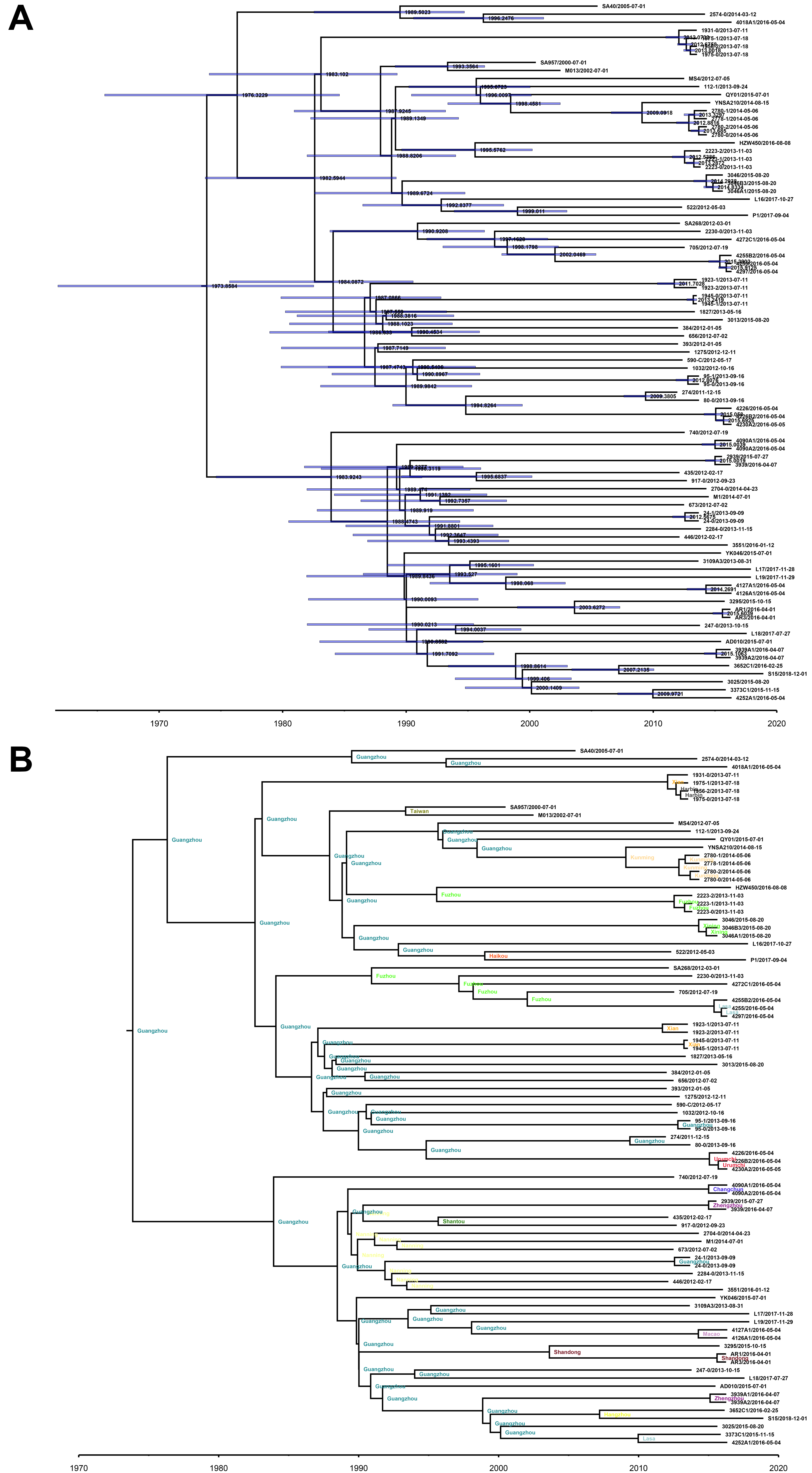

Supplement: Supplementary file 2 [file Image_2.TIF]

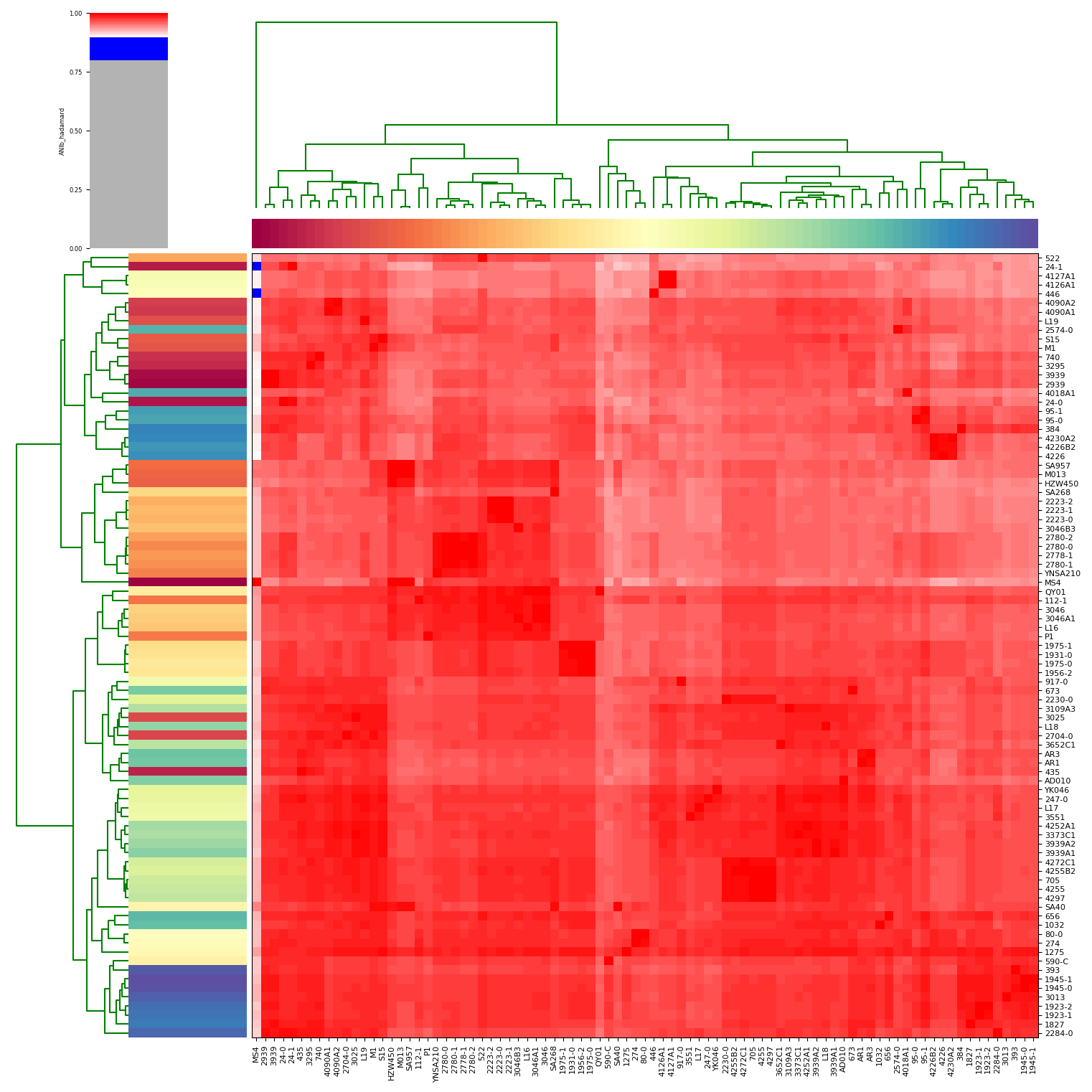

Supplement: Supplementary file 3 [file Image_3.TIF]
