# Supplementary material for: The Genomic Context for the Evolution and Transmission of Community-Associated Staphylococcus aureus ST59 Through the Food Chain
Source: Front Microbiol. 2020 Mar 17;11:422. doi: 10.3389/fmicb.2020.00422 (PMC7090029; doi:10.3389/fmicb.2020.00422)
Supplement: Supplementary file 9 [file Data_Sheet_1.DOCX]

**Supplementary Information of “The genomic context for the evolution and transmission of community-associated** ***Staphylococcus aureus* ST59 through the food chain”**

Rui Pang^a1^, Shi Wu^a1^, Feng Zhang^a^, Jiahui Huang^a^, Haoming Wu^a^, Junhui Zhang^b^, Yanping Li^a^, Yu Ding^b^, Jumei Zhang^a^, Moutong Chen^a^, Xianhu Wei^a^, Youxiong Zhang^a^, Qihui Gu^a^, Zhenwen Zhou^c^, Bingshao Liang^c^, Wenzhi Li^d^, Qingping Wu^a*^.

^a^ Guangdong Institute of Microbiology, Guangdong Academy of Sciences, State Key Laboratory of Applied Microbiology Southern China, Guangdong Provincial Key Laboratory of Microbial Culture Collection and Application, Guangdong Open Laboratory of Applied Microbiology, Guangzhou, China

^b^ Department of Food Science and Technology, Jinan University, Guangzhou, China

^c^ Clinical Laboratory, Guangzhou Women and Children’s Medical Center, Guangzhou Medical University

^d^ Infinitus (China) Company Ltd., Jiangmen, China

^*^ Corresponding author. E-mail address: [wuqp203@163.com](mailto:wuqp203@163.com) (Q. Wu).

^1^ These authors contributed equally to this work.

**Supplementary Information contains:**

Legends for Supplementary Figures 1 to 3.

**Figure S1.** The sampling site of foods sample in China.

**Figure S2.** The annotated Maximum clade credibility (MCC) tree representation of the BEAST analysis results of 81 *Staphylococcus aureus* CC59 isolates. (A) Node labels indicate divergence times of common ancestors of phylogenetic groups; the bars indicate the respective 95% highest posterior density intervals. (B) Node labels and colors indicate the isolates’ origins and allow the reconstruction of possible spatial transmission routes.

**Figure S3.** Hierarchical clustering in two dimensions of pairwise average nucleotide identity (ANI) comparison of CC59 isolates. The ANI values are presented as a heatmap generated according to the matrix of Hadamard product of pairwise alignment coverage, total alignment lengths, similarity errors, and percentage identity.
